# Identification of the 7-lncRNA Signature as a Prognostic Biomarker for Acute Myeloid Leukemia

**DOI:** 10.1155/2021/8223216

**Published:** 2021-12-20

**Authors:** Chun-yu Liu, Hui-hui Guo, Hai-xia Li, Ying Liang, Cong Tang, Nan-nan Chen

**Affiliations:** ^1^Department of Hematology, The No. 967 Hospital of PLA Joint Logistics Support Force, Dalian, Liaoning, China; ^2^Department of Obstetrics and Gynecology, The No.967 Hospital of PLA Joint Logistics Support Force, Dalian, Liaoning, China

## Abstract

A lot of evidence has emphasized the function of long noncoding RNAs (lncRNAs) in tumors' development and progression. Nevertheless, there is still a lack of lncRNA biomarkers that can predict the prognosis of acute myeloid leukemia (AML). Our goal was to develop a lncRNA marker with prognostic value for the survival of AML. AML patients' RNA sequencing data as well as clinical characteristics were obtained from the public TARGET database. Then, differentially expressed lncRNAs were identified in female and male AML samples. By adopting univariate and multivariate Cox regression analyses, AML patients' survival was predicted by a seven-lncRNA signature. It was found that 95 abnormal expressed lncRNAs existed in AML. Then, the analysis of multivariate Cox regression showed that, among them, 7 (LINC00461, RP11-309M23.1, AC016735.2, RP11-61I13.3, KIAA0087, RORB-AS1, and AC012354.6) had an obvious prognostic value, and according to their cumulative risk scores, these 7 lncRNA signatures could independently predict the AML patients' overall survival. Overall, the prognosis of AML patients could be predicted by a reliable tool, that is, seven-lncRNA prognostic signature.

## 1. Introduction

As a malignant and aggressive disease, acute myeloid leukemia (AML) has a lot of advantages, such as abnormal expansion of myeloid blasts, and it appears especially in the elderly [1, 2]. The cure rate of AML patients with an age of less than 60 is 20-35% higher than that of those over 60 years old [3]. Although AML patients have been treated with potential therapies (such as intensive chemotherapy and allogeneic stem cell transplantation), only a few are eligible for these treatments (because of treatment intolerance and lack of matched donors); for AML patients who receive transplants, more than 50% end up relapsing [4, 5]. Therefore, it is of great significance to explore effective biomarkers for the early detection and improvement of the prognosis of AML.

Nowadays, long noncoding RNAs (lncRNAs) have been paid more and more attention to in cancer research [6]. Meanwhile, lncRNAs are a class of nonprotein coding transcripts with a length of generally greater than 200 nucleotides [7]. In recent years, some researchers have proved that lncRNAs are indispensable through comprehensive mechanisms in a number of biological events, including cell differentiation, cell cycle, and apoptosis [8, 9]. Especially in the field of tumors, a larger number of researches have confirmed that lncRNAs are involved in tumor genesis and metastasis by multiple mechanisms, including sponging miRNAs, epigenetic regulation, translation regulation, cell differentiation regulation, and therapy resistance [10, 11]. It is noteworthy that these lncRNAs can be biomarkers as well as therapeutic targets for tumors. In this study, a seven-lncRNA model was established by using the Cox regression method, aiming to independently access prognosis and precisely predict survival probability in AML patients from the TARGET database.

## 2. Materials and Methods

### 2.1. Data Sources

The RNAseq data as well as their respective clinical follow-up material were downloaded from the public TARGET database [12], including 186 male AML samples and 172 female AML samples.

### 2.2. Differentially Expressed lncRNA Extraction and Analysis

The differentially expressed lncRNAs were estimated by using R statistical software package “limma.” ∣log(FC) | ≥0.5 and *p* value < 0.05 were used as cutoffs to screen dysregulated lncRNAs. Then the “ggpubr” as well as “pheatmap” packages produced boxplots and heat maps, respectively.

### 2.3. Risk Signature Construction and Validation

The definition of risk signature scores is Risk score = expression of LINC00461 × 0.097559 + expression of RP11 − 309 M23.1 × 0.087081 + expression of AC016735.2 × 0.107281 + expression of RP11 − 61I13.3 × 0.100018 + expression of KIAA0087 × 0.072299 + expression of RORB − AS1 × 0.081708 + expression of AC012354.6 × −0.24187. Then, the score was calculated in overall cohort, and the sample was divided into groups with high risk and low risk by using the median risk score. Through assessing the area under the curve (AUC), the “timeROC” R package was adopted to verify this immune-related risk signal's prognostic utility. Besides, the overall survival (OS) of patients with high and low risk was contrasted by the Kaplan-Meier curves as well as the “survival” R package [13].

### 2.4. Survival Analysis

lncRNAs whose expressions were correlated with AML patients' overall survival were recognized by multivariate Cox regression analysis. Meanwhile, genes whose *p* values are smaller than 0.05 were taken as candidate ones that are correlated with patients' survival. Moreover, the general survival was explored by the Kaplan-Meier survival curve, so as to evaluate the differences in patients' survival. More importantly, the receiver operating characteristic (ROC) curve (AUC) was used to prove the specificity for diagnostic accuracy, and then the cBioPortal tool was adopted to determine the alterations of the survival-associated lncRNAs in AML [14].

### 2.5. Statistical Analysis

According to the critical conditions of ∣log(FC) | ≥0.5 as well as an adjusted *p* value < 0.05, DELs were recognized by adopting the “edgeR” package [15] in the R software. Then, univariate and multivariate Cox regression analyses were implemented in order to recognize the lncRNAs with a prognostic value. The “timeROC” R package [16] was adopted to calculate the zone under the ROC curve (AUC), and Kaplan-Meier curves as well as log-rank tests “survival” R package were used to analyze patients' survival outcomes. *p* < 0.05 was considered to have a statistical significance, and all tests were two-sided. We used R (v.3.6.1, R Core Team, Boston, MA, USA) for the above data analysis.

## 3. Results

### 3.1. The Identification of Differentially Expressed lncRNAs in AML

**Figure 1:**
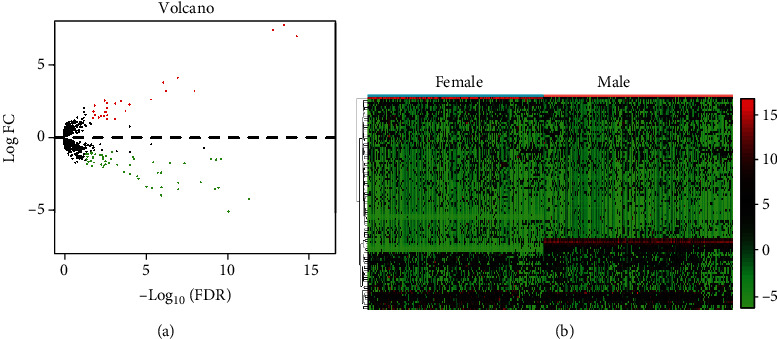
The expressing pattern of lncRNAs in AML. (a) Volcano plot of the differentially expressed lncRNAs in AML based on TARGET database. (b) Heat map of the differentially expressed lncRNAs in AML.

For the identification of dysregulated lncRNAs in AML patients, we downloaded microarray data from TARGET database and performed “R studio” software for statistical analysis. We found 95 abnormal expressed lncRNAs in AML, which were shown using volcano plot and heat map (Figures 1(a) and 1(b)).

### 3.2. The Identification of Prognostic lncRNAs in AML Patients

**Figure 2:**
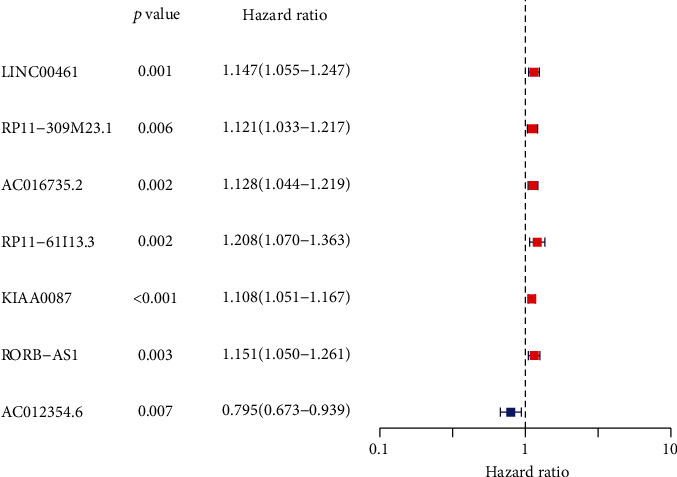
Multivariate Cox regression analysis confirmed the prognostic lncRNAs in AML.

Next, a multivariate Cox regression analysis was conducted to recognize the differentially expressed lncRNAs that were significantly related the AML patients' overall survival. It was found that a total of 7 of these differentially expressed lncRNAs were greatly related to the survival of AML patients (Figure 2).

### 3.3. Construction and Validation of Prognostic lncRNA Signals

**Figure 3:**
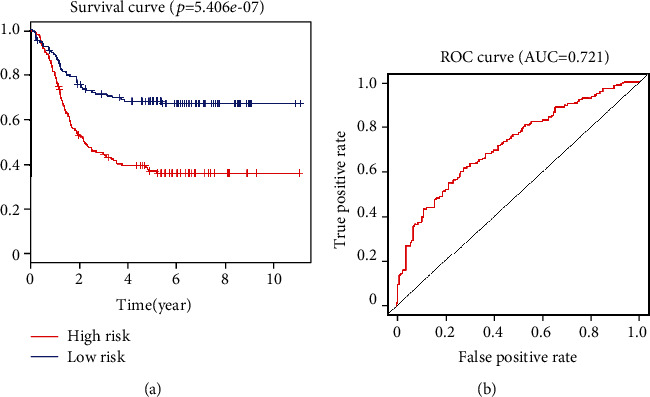
The construction of a prognostic risk signature related to outcomes of AML patients. (a) Low and high-risk AML patients' overall survivals were determined by the use of Kaplan-Meier curves. (b) Time-dependent ROC assays of the identified 7-lncRNA risk signature in AML patients.

**Figure 4:**
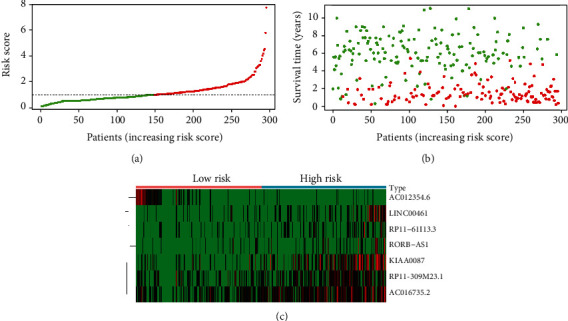
The characteristic of 7-lncRNA risk signature. (a) and (b) The distribution of risk scores, patient survival time, and status for overall survival. (c) The heat map of the seven prognostic lncRNAs in different risk groups.

The following prognostic model score was constructed by using these IRGs as well as their corresponding regression coefficient values: Risk score = the expression of LINC00461 × 0.097559 + that of RP11 − 309 M23.1 × 0.087081 + that of AC016735.2 × 0.107281 + that of RP11 − 61I13.3 × 0.100018 + that of KIAA0087 × 0.072299 + that of RORB − AS1 × 0.081708 + that of AC012354.6 × −0.24187. Six prognostic lncRNAs were associated with elevated risk (LINC00461, RP11-309M23.1, AC016735.2, RP11-61I13.3, KIAA0087, and RORB-AS1; Coef > 0), and one is a protective gene that was correlated with decreased risk (AC012354.6; Coef < 0) (Table 1). Then, the risk score method was adopted to provide scores for all samples, and the median risk score values were applied to divide patients into two groups, including low-risk (*n* = 148) as well as high-risk (*n* = 147) patients. Later, this risk scoring method is used to score all samples. The general survival of the low-risk group was obviously longer than that of the high-risk group (*p* = 5.406*e* − 07, Figure 3(a)), with a 3-year AUC value of 0.721 (Figure 3(b)). Besides, patients who had continuous risk scores had different clinical outcomes in different groups (Figures 4(a) and 4(b)). The expressing pattern of LINC00461, RP11-309M23.1, AC016735.2, RP11-61I13.3, KIAA0087, RORB-AS1, and AC012354.6 was shown in Figure 4(c).

## 4. Discussion

In the past few years, many nonprotein-coding transcripts of the genome, including lncRNAs, have been treated as irrelevant transcriptional junk [17]. Due to the success of ENCODE as well as the implementation of the Cancer Genome Atlas (TCGA) project, lncRNAs have attracted attention because of their important function in cancer occurrence and development [18, 19]. lncRNAs participate in a series of basic biological processes, such as cell cycle regulation, apoptosis, and DNA damage response, and their roles in some human diseases have been reported more and more [20, 21]. Recently, a number of researches have indicated that altered lncRNA expression levels were correlated with disease progression, but their prognostic value based on multiple models has rarely been studied [22–24].

In this study, we identified 95 dysregulated lncRNAs between 186 male AML patients and 172 female AML patients. Using multivariate Cox regression analysis, we identified 7 prognosis-related lncRNAs, including LINC00461, RP11-309M23.1, AC016735.2, RP11-61I13.3, KIAA0087, RORB-AS1, and AC012354.6. In recent years, several studies have reported the tumor-related function of LINC00461 in various tumors. For instance, LINC00461 was shown to strengthen the colorectal cancer cells' proliferation and invasion ability by miRNA-323b-3p/NFIB Axis [25]. LINC00461 was reported to be highly expressed in lung tumor, and its knockdown suppressed the tumor cells' proliferation and metastasis via miR-4478/E2F1 [26]. However, the expression and function of RP11-309 M23.1, AC016735.2, RP11-61I13.3, KIAA0087, RORB-AS1, and AC012354.6 were rarely reported. More experiments were needed to confirm their functions in various types of tumors.

In recent years, many studies have reported that lncRNAs have some potentials to be used as novel biomarkers for AML patients [27, 28]. For instance, it was reported that lncRNA ANRIL could be used to regulate the development of AML by regulating the AdipoR1/AMPK/SIRT1's glucose metabolism pathway [29]. Tao and his group showed that downregulating CD27-AS1 could inhibit proliferation and induce apoptosis of AML cells through the miR-224-5p/PBX3 [30]. In recent years, several prognostic models based on lncRNAs were developed in different types of tumors [31, 32]. However, at present, there are no reliable models based on critical lncRNAs that can predict prognosis in AML patients. In this study, the following prognostic model score was established by adopting the above 7 prognosis-related lncRNAs as well as their corresponding regression coefficient values, and it was found that high-risk patients displayed a generally shorter survival than low-risk ones, which suggested that this innovative lncRNA expression signature might be a powerful biomarker for the AML patients' prognosis.

However, there are several limitations to the present analysis. First, this study included 358 AML patients from TARGET database, which had a limited sample size. Second, our results' reliability is restrained because of a lack of in vitro or in vivo experiments. Third, as this research is retrospective, so a prospective study is needed to verify the findings of this study.

## 5. Conclusion

A 7-lncRNA risk signature correlated with AML prognosis is successfully constructed in the TARGET database. Our findings revealed that the signature is a potent predictive indicator for patients with AML.

## Figures and Tables

**Table 1:** Seven lncRNAs associated with the survivals of AML patients.

id	coef	exp (coef)	se (coef)	*z*	Pr (>∣*z*∣)
LINC00461	0.097559	1.102477	0.045227	2.157084	0.030999
RP11-309M23.1	0.087081	1.090985	0.04358	1.998193	0.045696
AC016735.2	0.107281	1.113247	0.039321	2.728368	0.006365
RP11-61I13.3	0.100018	1.105191	0.06338	1.578059	0.114552
KIAA0087	0.072299	1.074976	0.028702	2.518928	0.011771
RORB-AS1	0.081708	1.085139	0.04816	1.696594	0.089774
AC012354.6	-0.24187	0.785156	0.088975	-2.71844	0.006559

## Data Availability

The data used to support the findings of this study are available from the corresponding author upon request.
